# Heterotaxy Polysplenia Syndrome in Adulthood: Focused Review and a Case Report

**DOI:** 10.7759/cureus.6822

**Published:** 2020-01-30

**Authors:** Gustavo Lagrotta, Melanie Moises

**Affiliations:** 1 Internal Medicine, Larkin Community Hospital, Hialeah, USA

**Keywords:** heterotaxy syndrome, left isomerism, right isomerism, situs ambiguus, situs inversus

## Abstract

Heterotaxy syndrome (Situs ambiguus) is a condition in which the internal organs are abnormally arranged in the chest and abdomen. Individuals with this condition have complex birth defects affecting the heart, lungs, liver, spleen, intestines, and other organs. Unlike situs inversus, it often causes serious health problems. This report describes a case of a 49-year-old Hispanic female with a significant medical history of situs ambiguous diagnosed at birth in Cuba. She has had little to no follow-up in adulthood due to being “healthy.” She presented to the emergency room with intractable pain in the left lower quadrant and left flank for two days. Heterotaxy syndrome was found incidentally on CT scan of the abdomen/pelvis (plain). She was further evaluated with chest X-ray, magnetic resonance imaging of abdomen/pelvis without and with contrast, transvaginal ultrasonography, renal/bladder ultrasonography, left upper quadrant (LUQ) ultrasonography, esophagogastroduodenoscopy (EGD) with biopsy, 2D echocardiogram, and pertinent laboratory tests. Certain unusual findings included azygos continuation of the inferior vena cava (IVC), numerous spleens, atrophic pancreas, dilatation of duodenal C sweep, pelvic mass (possibly arising from right ovary), multiple nabothian cysts, and cardiac dysfunctions (such as severe mitral regurgitation). This report further aims to identify anatomic variants, previously established or otherwise not, in heterotaxy syndrome. Also, there seems to be a lack of identifiable anomalies or associations in regard to female anatomy, particular to this case being the female pelvic anatomy. As previous reports and research have stated, identification of anomalies in this syndrome is key for adequate and optimal management.

## Introduction

Heterotaxy syndrome (Situs ambiguus) is a condition in which the internal organs are abnormally arranged in the chest and abdomen. Individuals with this condition have complex birth defects affecting the cardiac, respiratory, gastrointestinal, genitourinary and other systems. Unlike situs inversus, it often causes serious health problems. It can alter the structure of the heart, including the attachment of the large blood vessels that carry blood to and from the rest of the body. It can also affect the structure of the lungs, such as the number of lobes in each lung and the length of the bronchi. In the abdomen, the condition can cause a person to have asplenia or multiple small, poorly functioning spleens (polysplenia). The liver may lie across the middle of the body instead of being in its normal position to the right of the stomach. The severity of heterotaxy syndrome varies depending on the specific abnormalities involved. Some affected individuals have only mild health problems related to the condition. At the other end of the spectrum, heterotaxy syndrome can be life-threatening in infancy or childhood, even with treatment [1]. The majority of patients with polysplenia syndrome die by age of 5 years. This high mortality rate is mainly due to severe cardiac abnormalities. Some 5-10% of patients with polysplenia syndrome have normal hearts or only minor cardiac defects and reach adulthood without symptoms, such as the one presented in this case [2].

There are two recognized variants of heterotaxy: left isomerism and right isomerism. Left isomerism is associated with paired left‐sided viscera, whereas right‐sided viscera may be absent. In contrast, right isomerism features paired right‐sided viscera, whereas left-sided viscera may be absent. Both variants are associated with complex cardiac malformations. Typical findings in left isomerism are bilateral morphologic left atrial appendages (left atrial isomerism), viscerocardiac heterotaxy (situs ambiguous, with incoherent laterality of the heart axis, stomach, portal sinus, or gallbladder), multiple cardiac anomalies (with a predominance of atrioventricular septal defects and pulmonary stenosis), congenital heart block, bilateral morphologic left (bilobed) lungs with hyparterial bronchi, multiple splenuli (polysplenia), intestinal malrotation, nonrandomly genitourinary malformations, and interruption of the inferior vena cava (IVC) with azygos continuation [3]. This case report presents a 49-year-old Hispanic female with a significant medical history of situs ambiguous diagnosed at birth in Cuba, who can now classify under the left isomerism variant given the radiological findings.

## Case presentation

The patient is a 49-year-old Hispanic female with a significant past medical history of situs ambiguous diagnosed at birth in Cuba. She presented to the emergency room with a chief complaint of intractable pain in the left lower quadrant and left flank for two days. Pain was described as a stabbing and intermittent lasting as long as 20 minutes per episode a few times per day. She denied previous episodes of similar pain presentation, association with food or activity, or other symptoms such as nausea, vomiting, diarrhea, constipation, and problems urinating.

In addition to situs ambiguous, the patient has a history of asthma. She has not had the need to establish a primary care physician as she states she has been in good health all of her life. She denies use of home medications, vitamins, or supplements. Surgical history is significant for intussusception repair at two days old. She was seen at the emergency room two weeks prior for shortness of breath but was not admitted. The patient is an immigrant from Cuba and currently unemployed. She smokes one pack per day for the last 25 years. Her mother has hypertension and diabetes and her father’s medical history is unknown. She has one healthy child (G1P1) via natural birth.

On physical examination, vital signs were all within normal limits: regular rate and rhythm and grade III holosystolic murmur. A 5-cm surgical midline scar was visualized. Hypoactive sounds were noted in all quadrants. Guarding was present with tenderness to palpation in left upper and lower quadrants. Negative costovertebral angle (CVA) tenderness, rebound tenderness, and rigidity.

On admission, relevant laboratory findings only included urinalysis with microscopic hematuria at 10-20 red blood cells (RBC) per high-power field (HPF). Basic metabolic profile, complete blood cell count, liver profile, and lipase were unremarkable. Pregnancy serum qualitative test was negative.

**Figure 1:**
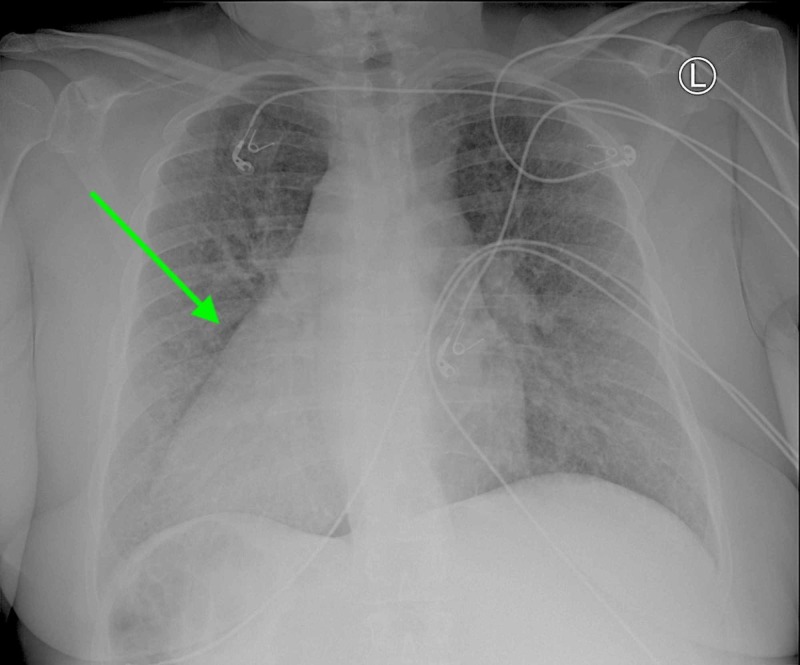
Anterior-Posterior chest X-ray demonstrating dextrocardia.

**Figure 2:**
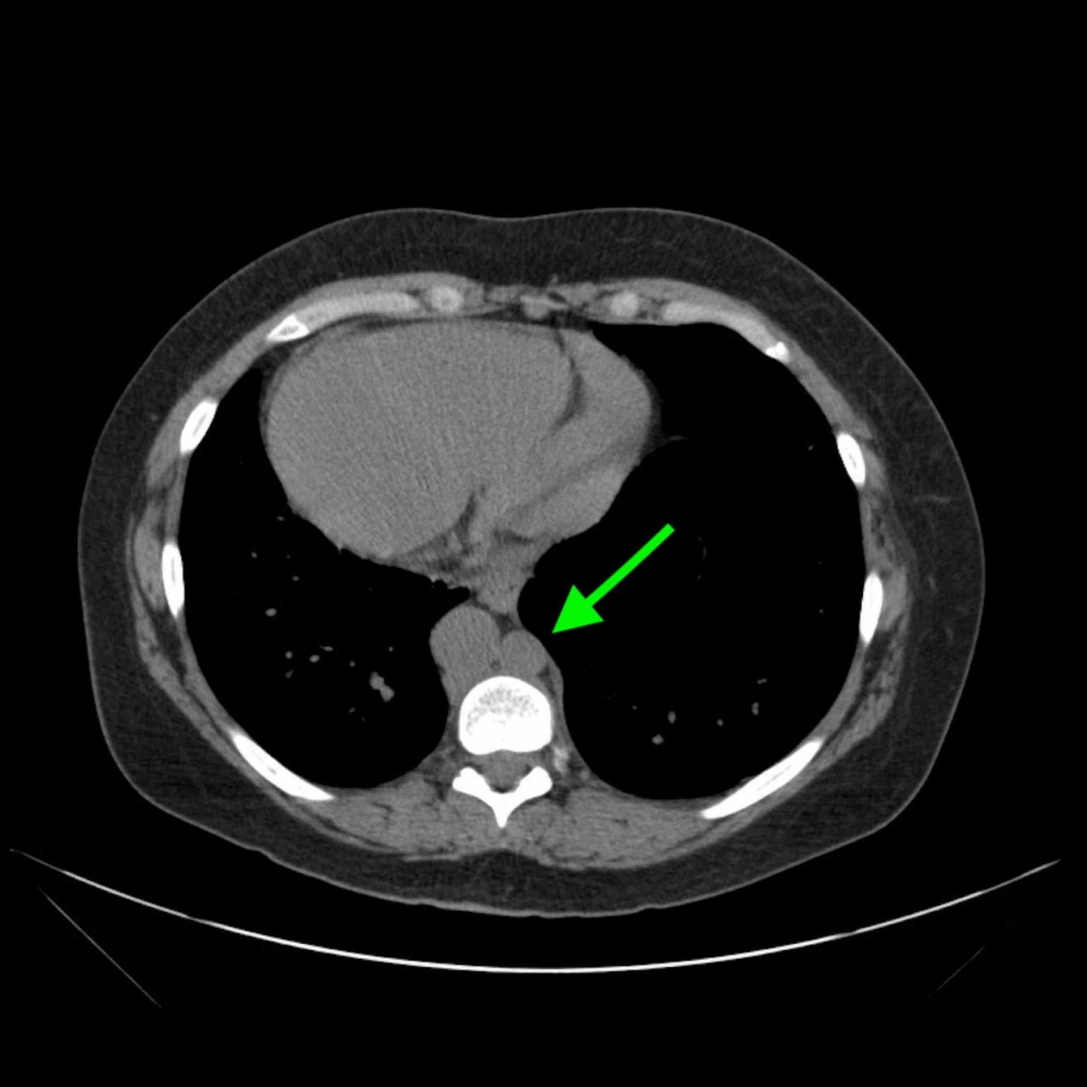
Inferior vena cava with azygos vein continuation on abdominal CT without contrast.

**Figure 3:**
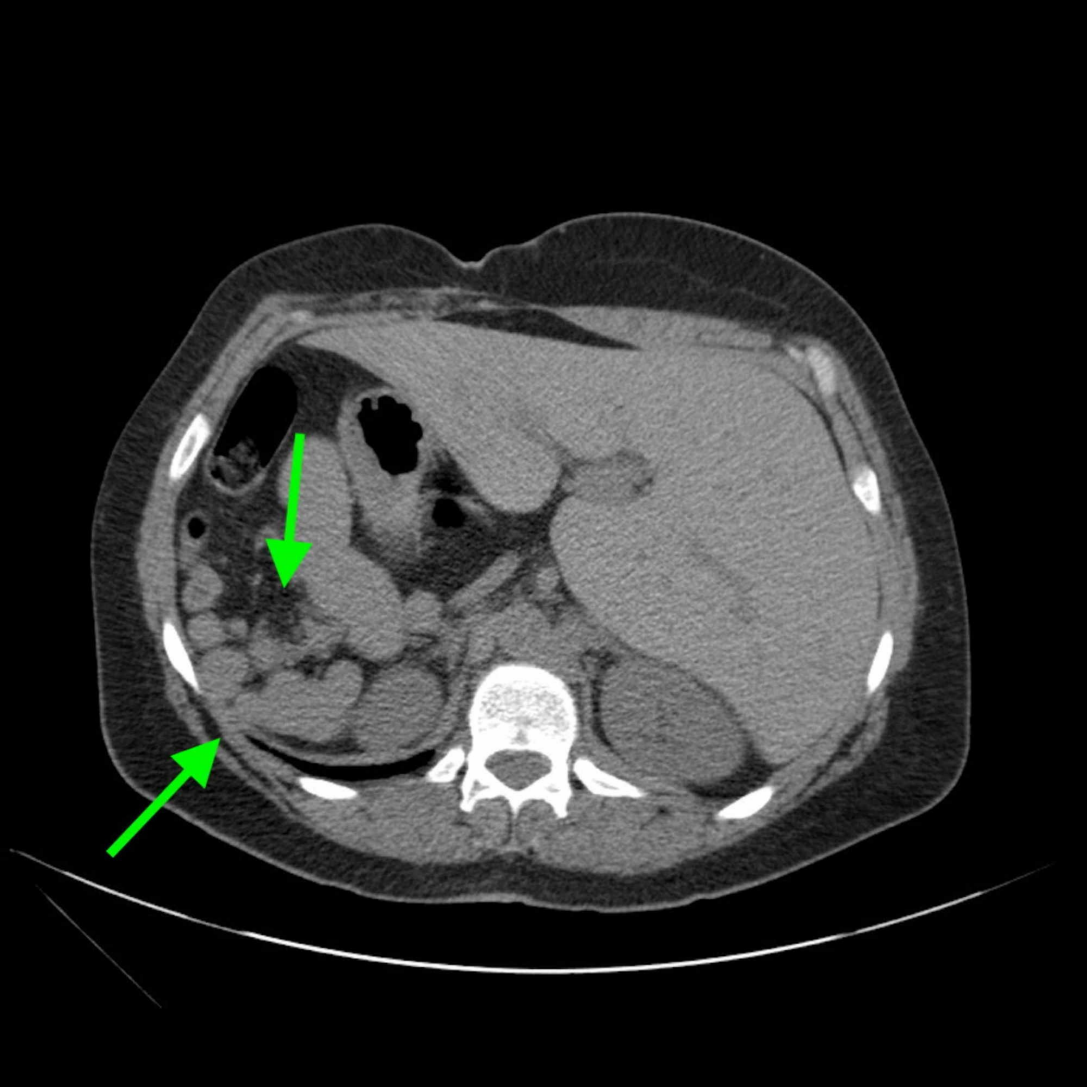
Splenules noted CT abdomen and pelvis.

Chest X-ray and CT abdomen and pelvis (plain) were performed on admission. Chest X-ray demonstrated prominent interstitial markings suggestive of chronic vs. acute interstitial disease, as well as a cardiomediastinal silhouette within normal limit size and a cardiac apex pointing to the right (Figure 1). CT showed situs ambiguous with cardiac apex and stomach on the right, liver on the left, multiple spleens, azygos continuation of IVC (Figure 2). It also showed a 1.6-cm cystic lesion with mural nodule in the cervix and an 8.0-cm cyst extending from the right aspect of the uterus near the midline of the pelvis causing mass effect on the bladder (Figure 3).

Ultrasound (US) renal/bladder was obtained due to microscopic hematuria, which revealed unremarkable bilateral kidneys, and no abnormal bladder masses or ureteral calculi.

**Figure 4:**
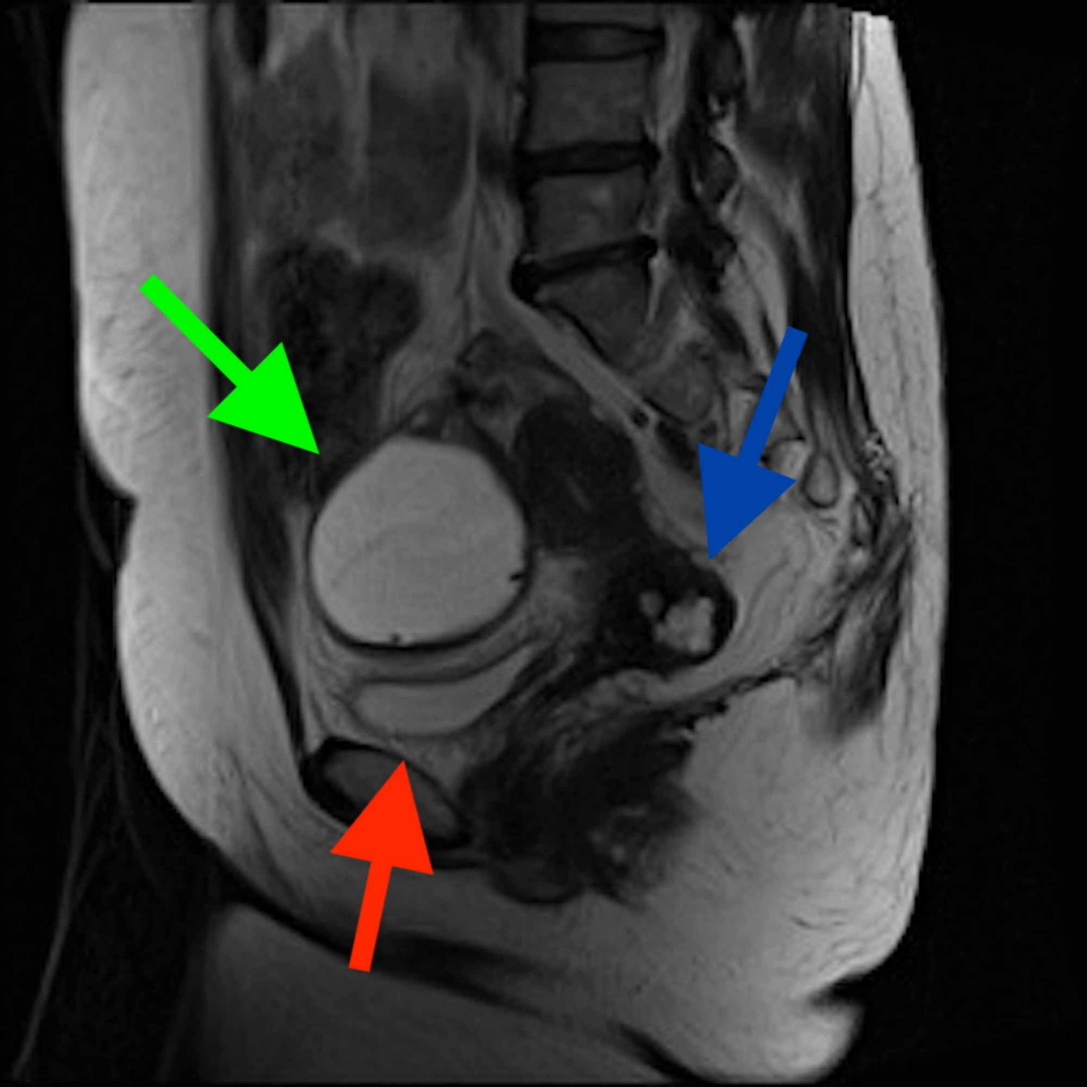
MRI sagittal plane view of ovarian cyst (green arrow) causing mass effect on bladder (red arrow). Nabothian cysts (blue arrow) also noted.

**Figure 5:**
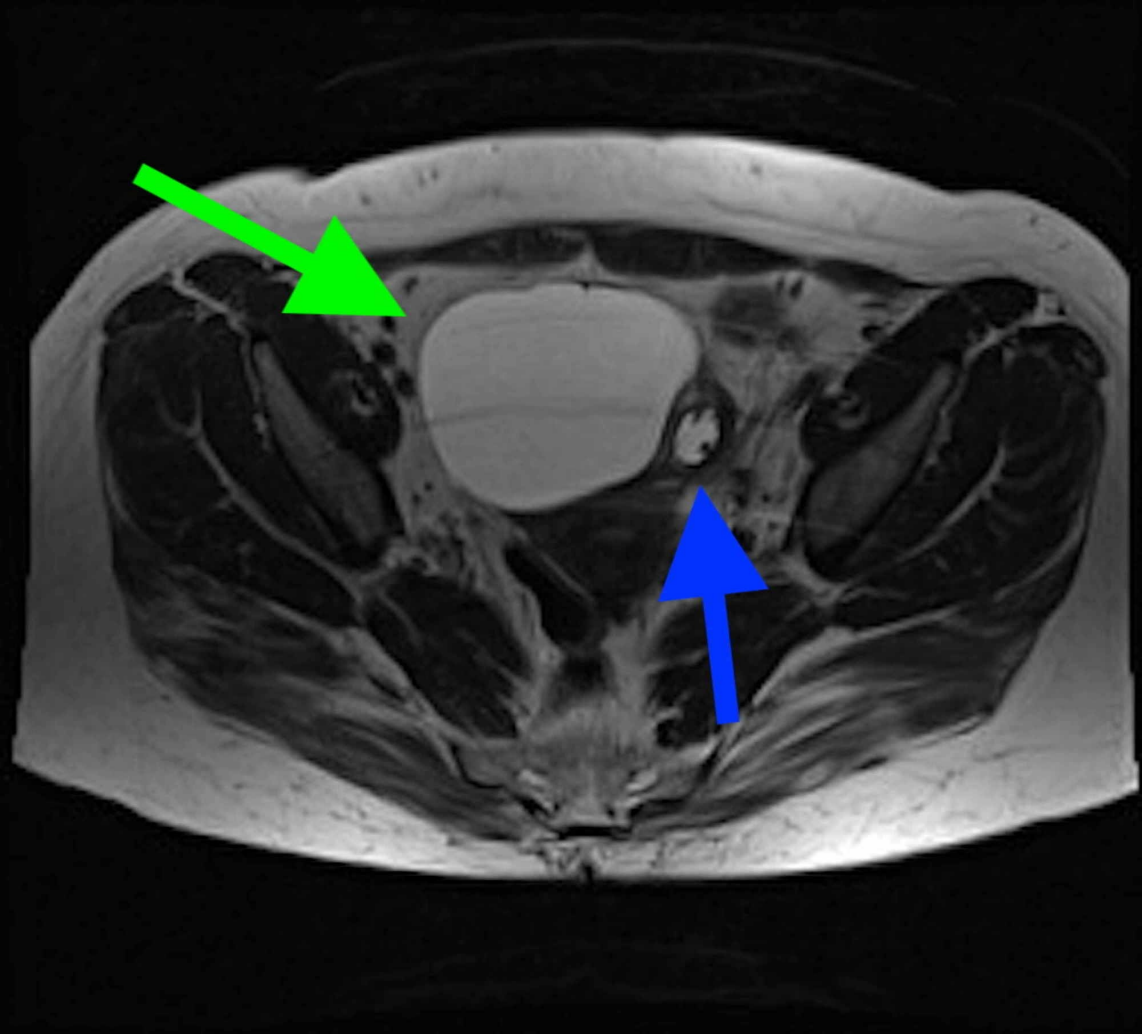
MRI transverse plane view of ovarian cyst (green arrow) causing mass effect on bladder. Nabothian cysts (blue arrow) also noted.

CT findings justified further evaluation with MRI abdomen/pelvis without and with contrast. The MRI of the pelvis showed an anteverted uterus normal in size with a non-thickened endometrium. There were several small 6 to 12 mm simple fluid intensity cysts compatible with nabothian cysts. There was a round, thin-walled fluid intensity mass measuring 7 x 8.4 x 7 cm with thin enhancing internal septa (possible serous cystadenoma vs. ovarian cyst), possibly arising from the right ovary, which was causing mass effect on the urinary bladder (Figures 4, 5). No enlarged or bulky retroperitoneal lymph nodes were seen. In the abdomen, intra-abdominal organs were consistent with situs inversus. The pancreas was atrophic in size, and the spleen was small in size with multiple adjacent splenules/accessory spleens. There was dilatation of the duodenal C sweep (located on the left side) up to 6.4 cm with appearance of a transition point at the junction of the 2nd and 3rd portions of the duodenum, suspicious for focal narrowing and partial obstruction.

An esophagogastroduodenoscopy (EGD) was performed to evaluate for possible narrowing and obstruction. It revealed compression of the 2nd portion of the duodenum, retained food products in the first portion of the duodenum. A biopsy showed mild chronic gastritis, negative for intestinal metaplasia, and negative for H. pylori microorganisms. Also, a small bowel series was ordered but the patient could not tolerate barium.

**Figure 6:**
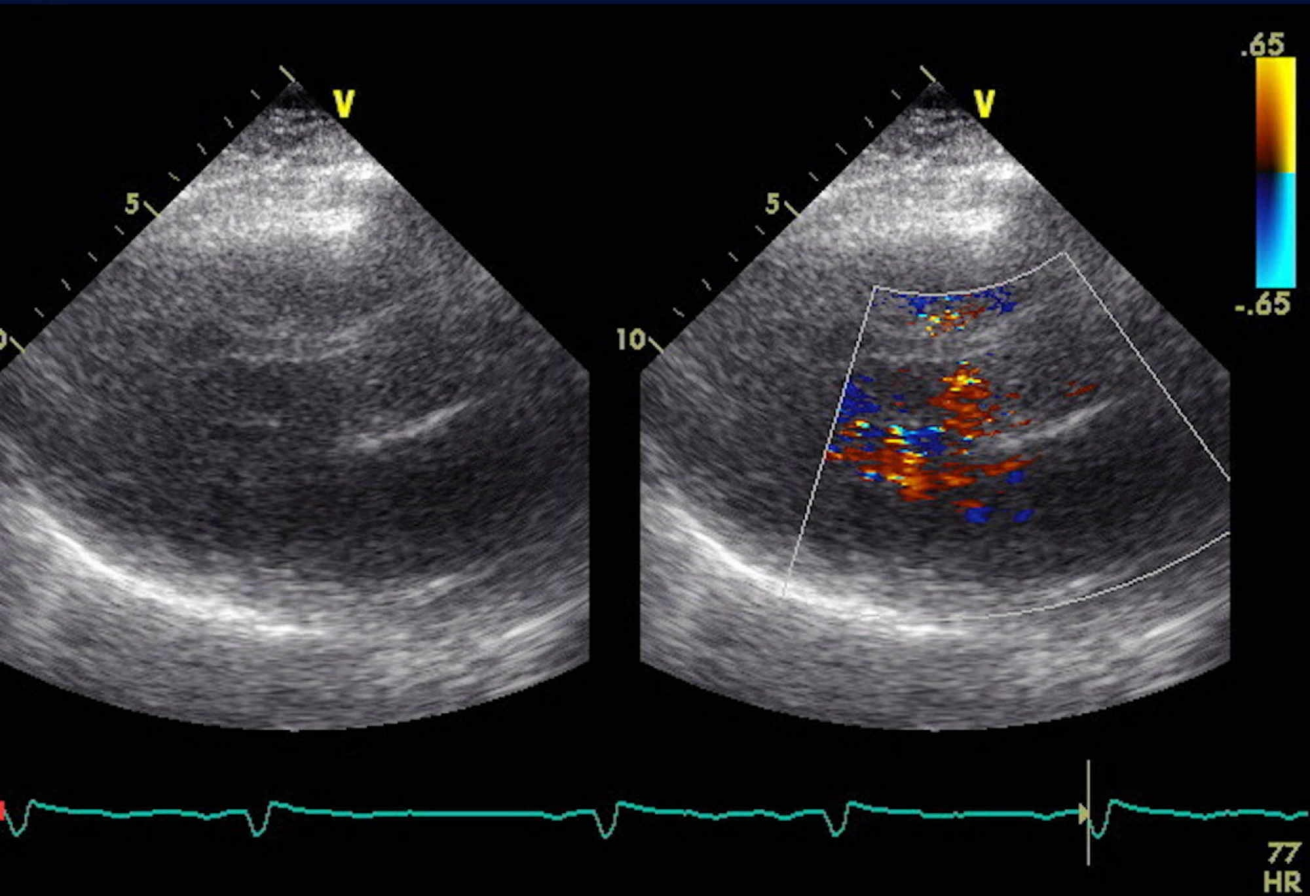
2D echocardiogram.

**Figure 7:**
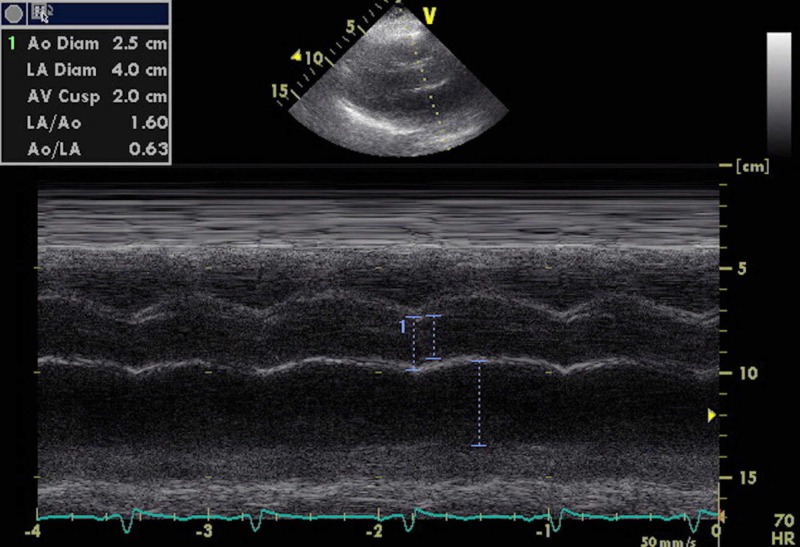
2D echocardiogram.

A 2D echocardiogram from her previous visit was reviewed (Figures 6, 7). Preserved ejection fraction at 60%, severe mitral regurgitation, mild atrial stenosis, pseudonormal diastolic function, and enlargement of the left atrium. No other anatomic cardiac anomalies were identified.

In order to rule out the presence of a cystadenoma, CA 125 was obtained and found to be 16.3 (0.0-38.1). Other cancer serological marker obtained were CA 15-3 at 14.3 (0.0-25) and CA 19-9 at 4 (0-35).

The patient was placed NPO (nil per os) and treated conservatively with intravenous fluids and Miralax. She had several bowel movements, which led to resolution of her initial pain. She was subsequently discharged and advised to follow up with a primary care physician to establish care and referral to a proper multidisciplinary team.

## Discussion

Heterotaxy syndrome is quite uncommon in the population. Figures for situs ambiguus provided by The Baltimore-Washington Infant Study estimated 1.44/10,000 incidence for all cardiac defects associated with left-right asymmetry malformations [4]. These figures might be an underestimation since there is a percentage of patients that go without diagnosis due to lack of clinical manifestations. This has made it challenging to document and record cases of this syndrome, especially in adulthood. In left isomerism children may have septal defects, heart conductivity issues leading to complete heart block, asplenia or polysplenia, and midline structure malformations including the gastrointestinal tract such as gut malrotation and biliary atresia [5, 6]. Although patients with left isomerism have a better prognosis than those with right isomerism, mortality and morbidity remain a point of concern [3]. Patients with complex cardiac lesions have a one-year mortality of 85% if they present with asplenia and greater than 50% if they present with polysplenia [7]. Early surgical intervention is critical for those within this population that have substantial cardiac and gastrointestinal abnormalities as it improves early survival, but despite interventions, late morbidity and mortality remains high [8]. Therefore, data in the adult population is scarce. This particular patient falls within the population who underwent surgical intervention at birth for a likely volvulus. From her echocardiogram findings and history, she does not fortunately present with any debilitating cardiac malformations. She has managed to conduct a healthy lifestyle, and close follow-up is warranted to better understand the complexities of this syndrome.

From a cardiological standpoint, left isomerism, the sinus and atrioventricular nodes are right atrial structures. These patients often have sinus node dysfunction or congenital heart block [9]. Our patient did present with mild cardiac anomalies, but none of which were significant. However, the importance of frequent follow-up with a multidisciplinary team should be reinforced in these patients. With strong associations present between this syndrome and conductivity issues, one can suspect patients are at a higher risk of a cardiovascular event.

Our patient also presented with an interrupted IVC with azygos continuation and polyspenia. Polysplenia is noted in 2.5:100,000 live births and it is present in 55% of patients with left isomerism [9,10]. It is often seen in conjunction with an interrupted IVC with azygos vein such as this particular case. This might be sometimes the only presentation in heterotaxy syndrome, and in such occasions, there are no clear complications that arise from it, hemodynamically or otherwise [11]. However, there is no concrete data or protocol of how these patients should be managed in the event of surgical interventions or health maintenance.

Furthermore, several genitourinary abnormalities have been reported, including kidneys and ureters, and even testes [11, 12]. But, once again the data is scarce for sex specific anomalies. There is no strong associations or presentations in the female anatomy. This patient presented with multiple nabothian cysts and a large ovarian cyst causing mass effect on the bladder. Further testing was done in order to rule out the possibility of malignancy. This raises the following question: Should patients with heterotaxy syndrome, or similar presentations, follow the same screening and health maintenance guidelines as the general population? We are aware that some of these patients go undiagnosed, and with medical advancements the mortality and morbidity will eventually decrease. Thus, patients should be monitored to a closer extent as there is no significant data at present time detailing any associations beyond those secondary to anatomic anomalies.

## Conclusions

As heterotaxy syndrome remains an uncommon presentation, more data is necessary in order to provide ideal medical care to this patient population. As mortality and morbidity decreases within this population and more people can reach adulthood, it is crucial for the primary general providers to provide close watch and build a multidisciplinary team around these patients for optimal care.
